# Association Between Hypercholesterolemia and Lumbar Degenerative Back Pain: A Medicare Expenditure Panel Survey (MEPS) Study

**DOI:** 10.7759/cureus.47930

**Published:** 2023-10-29

**Authors:** Idris Hanidu, Ryan Johnson, Peter Ahorukomeye, Nicholas U Ahn

**Affiliations:** 1 Orthopedic Surgery, University of Illinois at Chicago, Chicago, USA; 2 Orthopedic Surgery, Meharry Medical College School of Medicine, Atlanta, USA; 3 Orthopedic Surgery, Case Western Reserve University School of Medicine, Cleveland, USA; 4 Orthopedic Surgery, University Hospitals Cleveland Medical Center, Cleveland, USA

**Keywords:** back-pain, hypercholesterolemia, degenerative lumbar disease, lumbar spine, spine

## Abstract

Introduction

Hypercholesterolemia is known to be a major contributor to the morbidity associated with cardiovascular disease and has been hypothesized to result in degenerative changes to the spine through atherosclerosis of segmental lumbar vessels. The purpose of this study is to determine the relationship between hypercholesterolemia and degenerative lumbar spine conditions in a U.S. cohort.

Methods

A total of 30,461 participated in the 2018 Medicare Expenditure Panel Survey (MEPS). Of those, 1,063 subjects responded to whether a diagnosis of lumbar disorders with low back pain was present. Odds ratios (OR) were calculated, and logistic regression analyses were adjusted for demographic, education, occupation, cardiovascular and mental health conditions.

Results

Of the 1,063 respondents, 455 (43%) reported back pain. Mean age of the respondents was 62.7±16.1. Men and women reported back pain at similar rates (43% vs 45%, p=0.664). Age, race, education level and occupation were similar between those with and without back pain (p>0.05). Those with a diagnosis of depression had higher odds of having back pain (p<0.05). Prevalence of back pain in subjects who responded to the back pain diagnosis item on the survey was 42.6%. On univariate analysis, diagnosis of total cholesterol levels was significantly higher in those with a diagnosis of back pain (OR 1.36, 95% CI [1.20-1.54], p<.0001). Multivariable analysis showed that hypercholesterolemia was independently associated with back pain (adjusted OR 1.32, 95% CI [1.04-1.68], p=0.021) after controlling for covariates.

Conclusions

In this study, subjects with hypercholesterolemia were 34% more likely to have back pain after controlling for confounders which presents as a recent discovery amongst U.S. populations. Further studies should be performed to investigate the management of hypercholesterolemia in the development and progression of degenerative lumbar back pain.

## Introduction

Back pain is one of the most ubiquitous conditions affecting adults in the United States [1]. Evidence of lumbar intervertebral disc degeneration is present in nearly all adults with back pain [2]. Despite its prevalence, there are still various hypotheses for the etiology of this condition. The vascular theory proposes that diminished blood flow to the lumbar spine results in degenerative changes to the intervertebral discs, culminating in back pain [3-7]. Blood supply to the lumbar spine is precarious at baseline [3,8,9]. With advancing age, anastomotic networks between adjacent vertebrae are less robust, and thus blood supply to the lower lumbar vertebrae is diminished [8]. Atherosclerotic plaques can further limit the blood supply to the vertebrae and intervertebral discs. Several studies have shown associations between lumbar degenerative disease reflecting back pain and atherosclerosis of the lumbar segmental arteries [3-6,8,9]. It has thus been hypothesized that back pain is a result of ischemic injury to the lumbar discs, which at baseline have a tenuous blood supply [3-5]. 

The presence of high cholesterol has previously been implicated in the pathogenesis of disc degeneration [5,6,8,9]. However, these prior studies were performed on postmortem cadaveric subjects. Few population-based studies, including the Framingham study by Kauppila et al., have examined the relationship of atherosclerotic and cardiovascular risk factors as they relate to low back pain and lumbar degenerative disease [3]. 

This epidemiological study aims to conduct a retrospective cross-sectional analysis using the Medicare Expenditure Panel Survey (MEPS) data in a U.S. representative population, adjusting for relevant covariates in assessing the relationship between hypercholesterolemia and back pain. The results may provide supportive evidence for the vascular hypothesis for lumbar back pain.

## Materials and methods

Patient population and sampling

A retrospective cross-sectional analysis of the 2018 Medicare Expenditure Panel Survey (MEPS) was performed [3]. MEPS is a nationally representative survey of U.S. adults using a multistage, stratified sampling procedure conducted annually by the Agency for Healthcare Research and Quality (AHRQ). The MEPS Household component has pooled full-year consolidated data from a new panel of sample households each year from the National Health Interview Survey. There are several rounds of interviewing for families and individuals covering two calendar years. Data were obtained from their website (http://meps.ahrq.gov). This protocol was exempt from our institutional review boards due to its de-identified nature. 

A total of 30,461 participated in the 2018 MEPS. We focused our analysis on those who completed the back pain diagnosis items in the questionnaire [3]. Of the total cohort, 1,063 respondents answered the back pain items and 22,725 participants responded to the high serum cholesterol diagnosis items in the questionnaire [3].

Covariates

Participant-specific socioeconomic and lifestyle characteristics included age, sex, race, Body Mass Index (BMI), diabetes mellitus, hypertension, smoking habits, alcohol abuse, income, education and occupation. Age was measured in years and subcategorized as (a) 20-40, (b) 40-60) and (c) >60 years of age. Race was dichotomized into white and non-white groups. BMI was measured in kg/m2 and categorized according to Centers for Disease Control and Prevention (CDC) [10] classifications as underweight (<18.5), normal weight (18.5-24.9), overweight (25-29.9) or obese (≥30). Education level was measured in years. Total family income was measured in U.S. dollars. Smoking habits had three categories: (a) every day, (b) some days, and (c) not at all [3]. Occupation was re-grouped into eight groups: (a) Management, Business, and Financial Operations (abbreviated Management, Business and Fin. Op.), (b) Professional and related occupations, (c) Service occupations, (d) Sales and related occupations, (e) Office and administrative support, and (f) Construction, extraction, and Maintenance. Information on comorbidities was assessed by whether or not they currently had a diagnosis. Mental health information was also ascertained as part of the household survey [3]. All variables were self-reported using a standardized questionnaire.

Statistical analysis 

Descriptive statistics for continuous variables were calculated as means ± standard deviation, categorical variables as frequency and percentages (%) [3]. Differences in the characteristics by back pain diagnosis status were assessed with independent t-tests, ANOVA and chi-square tests when appropriate for continuous variables and categorical variables [3]. The association between hypercholesterolemia and back pain was assessed using logistic regression analysis for complex sampling adjusted for select variables [3]. This was represented as odds ratios (OR) with 95% confidence intervals (CI). A threshold of p<0.05 was established to achieve statistical significance [3]. All statistical methods were performed using JMP®, Version 16.0.0 (SAS Institute Inc., Cary, NC, USA) [3]. Only variables under the convariate section that reached statistical significance (p<0.05) were selected for multivariate analysis to assess the correlation between prevalence and outcomes with respect to back pain. All aforementioned variables under the covariate section that exhibited non-significance (p>0.05) were thoroughly documented and excluded from the study results due to non-adherance to study reliability.

## Results

A total of 30,461 subjects responded to the MEPS 2018 survey. Of those, 1,063 completed the Back Pain items and were included in the analysis. Mean age of the respondents was 62.7±16.1 (range 20-86) years. Mean age of those diagnosed with high serum cholesterol was 67.5±12.4 (range 29-85). The overall prevalence of back pain among respondents was 42.6%. The prevalence of back pain among males (43%) and females (45%) was comparable (p=0.612). Half of the underweight and obese individuals had back pain, while prevalence of back pain in normal weight was the lowest amongst the groups (16%) (p<0.05) [3]. Back pain was more prevalent in subjects with depression than without (p<0.05). There was a higher prevalence of back pain amongst those with high serum cholesterol levels (58% vs 45%, p<0.05) (Table 1) [3].

**Table 1:** Baseline Demographic Information IQR: interquartile range

	Back Pain		
	Yes (n=455)	No (n= 608)	p-value
Age (mean ± SD)	62.3±15.2	63.0±16.7	0.496
Sex			0.664
Male	412 (91%)	556 (91%)	
Female	43 (9%)	52 (9%)	
Race			0.633
White	367 (81%)	498 (82%)	
Other	88 (19%)	110 (18%)	
BMI			<0.05
Underweight	7 (2%)	7 (1%)	
Normal weight	73 (16%)	155 (25%)	
Overweight	169 (37%)	226 (37%)	
Obese	174 (38%)	176 (29%)	
Smoking habits			0.160
Everyday	68 (15%)	67 (11%)	
Some days	14 (3%)	21 (3%)	
Not at all	373 (82%)	520 (86%)	
Total family income (Median, IQR) ($)	61,798 (30,000–113,154)	66,408 (36,605–112,479)	0.257
Education (years)	13.8±2.2	13.9±2.2	0.605
Occupation			0.519
Management, Business, and Fin. Op.	39 (9%)	57 (9%)	
Professional and related	31 (7%)	59 (10%)	
Service occupations	20 (4%)	48 (8%)	
Sales and related	17 (4%)	22 (4%)	
Office and administrative support	11 (2%)	30 (5%)	
Construction, extraction, and Maintenance	15 (3%)	31 (5%)	
Production, transportation and material moving	29 (6%)	46 (8%)	
Alcohol abuse diagnosis	26 (6%)	25 (4%)	0.185
Depression diagnosis	126 (28%)	66 (11%)	<0.05
High cholesterol	263 (58%)	272 (45%)	<0.05

Back pain exposure was analyzed based on a univariate analysis to determine its prevalence against controlled variables. On univariate analysis, back pain was more prevalent in the 40-60 years age group (OR 1.25, 95% CI [1.02-1.52], p=0.029). Hypertension had slightly higher odds of back pain (OR 1.18, 95% CI [1.04-1.34], p=0.008). Depression was also significantly more prevalent in those with back pain (OR 1.80, 95% CI [1.53-2.12], p<0.0001) [3]. Back pain had significantly higher odds in those with hypercholesterolemia (OR 1.36, 95% CI [1.20-1.54], p<0.0001) (Table 2) [3]. These variables were then selectively adjusted into multivariable model that showed hypercholesterolemia was independently associated with an increased odds of back pain (selectively adjusted OR 1.34, 95% CI [1.16-1.54], p<0.0001) [3]. The fully adjusted model also had a higher prevalence of back pain (OR 1.32, 95% CI [1.04-1.68], p=0.021) (Table 3). 

**Table 2:** Univariate Analysis OR: odds ratio

	OR (95% CI)	p-value
Age (years)		
20-40	Reference	
40-60	1.25 (1.02–1.52)	0.029
>60	0.96 (0.80–1.14)	0.611
Sex (male)	0.95 (0.77–1.17)	0.612
Race		
White	Reference	
Non-white	1.04 (0.89–1.22)	0.605
BMI		
Normal weight	Reference	
Underweight	1.30 (0.58–2.93)	0.514
Overweight	0.97 (0.71–1.34)	0.867
Obese	1.29 (0.93–1.77)	0.119
Smoking habits		
Not at all	Reference	
Everyday	1.29 (0.94–1.79)	0.118
Some days	0.85 (0.52–1.35)	0.490
Total family income	1.00 (1.00–1.00)	0.685
Education (years)	0.98 (0.93–1.03)	0.396
Occupation		
Management, Business, and Fin. Op.	1.27 (0.85–1.90)	0.239
Professional and related	0.98 (0.64–1.48)	0.917
Service occupations	0.78 (0.47–1.25)	0.306
Sales and related	1.44 (0.80–2.55)	0.215
Office and administrative support	0.68 (0.35–1.24)	0.227
Construction, extraction, and Maintenance	0.90 (0.50–1.56)	0.714
Diabetes	1.05 (0.90–1.22)	0.506
Hypertension	1.18 (1.04–1.34)	0.008
Alcohol abuse diagnosis	1.19 (0.89–1.58)	0.238
Depression diagnosis	1.80 (1.53–2.12)	<0.001
Hypercholesterolemia	1.36 (1.20–1.54)	<0.001

**Table 3:** Multivariate Analysis OR: odds ratio

	Selectively adjusted		Fully adjusted	
	OR (95% CI)	p-value	OR (95% CI)	p-value
Age (years)				
40-60	1.18 (0.96–1.46)	0.116	1.15 (0.86–1.53)	0.341
>60	0.87 (0.71–1.07)	0.192	0.74 (0.52–1.05)	0.097
Sex (Male)			0.78 (0.56–1.08)	0.136
Race (Other)			0.99(0.75–1.29)	0.919
BMI				
Underweight			0.91 (0.09–5.94)	0.925
Overweight			1.04 (0.52–2.39)	0.915
Obese			1.23 (0.62–2.81)	0.561
Education level			1.01 (0.91–1.14)	0.846
Total Family Income			1.00 (1.00–1.00)	0.752
Occupation				
Management, Business, and Fin. Op.			1.47 (0.92–2.34)	0.102
Professional and related			0.85 (0.52–1.38)	0.527
Service occupations			0.75 (0.42–1.27)	0.293
Sales and related			1.58 (0.83–2.94)	0.156
Office and administrative support			0.61 (0.29–1.21)	0.170
Construction, extraction, and Maintenance			0.93 (0.48–1.77)	0.837
Smoking habits				
Everyday			1.04 (0.59–1.87)	0.895
Somedays			0.87 (0.34–1.98)	0.742
Diabetes			0.75 (0.53–1.06)	0.107
Hypertension	1.09 (0.94–1.25)	0.255	1.10 (0.86–1.39)	0.451
Alcohol abuse			1.29 (0.78–2.14)	0.319
Depression	1.70 (1.43–2.02)		1.46 (1.09–1.95)	0.010
Hypercholesterolemia	1.34 (1.16–1.54)	<0.001	1.32 (1.04–1.68)	0.021

## Discussion

Our study utilizes a large-scale nationally representative sample to demonstrate that high cholesterol is independently associated with back pain in American adults. Subjects with high serum cholesterol are at a 34% greater odds of having low back pain after controlling for relevant patient-specific, lifestyle, socioeconomic and mental health factors. 

Although prior studies have shown an association between cholesterol and lumbar pain disorders, there is a paucity of studies in a representative sample involving the North American population. A large-scale prospective study with 16-year follow-up by Jhawar et al. is the only U.S. study on female nurses that found the risk for disc herniation was 26% greater in those with high serum cholesterol levels when compared to those with normal serum cholesterol [11]. However, this study specifically examined the presence of lumbar disc herniations, which may have been asymptomatic, and not back pain in general. As such, only 0.8% of data points were found to have the presence of such a lesion.

There have been a few cross-sectional studies in other populations as well [12-14]. Hangai et al. studied 270 elderly Japanese patients and assessed the relationship between hypercholesterolemia and disc degeneration on MR images [14]. Their findings showed that those with high low-density lipoprotein (LDL) cholesterol had increased odds of L4/5 disc degeneration. However, this study encompassed only a small sample of fewer than 300 individuals, and only used radiographic MR findings, as opposed to clinical symptoms of back pain, as the outcome variable. As some element of disc degeneration is very common in adults over the age of 50, especially at the L4/5 level, whether the data from this study was clinically significant remained questionable [14]. 

Other studies, such as those of Heuch et al. [13] and Yoshimoto et al. [15], showed an association between back pain and high-density lipoprotein (HDL) cholesterol levels. While these studies encompassed large sample sizes, they did not specifically correct for many covariates such as occupation, income, and mental health conditions, all of which have been shown to significantly impact the presence of low back pain in prior studies [16-20].

A series of works by Kauppila and a few others revealed the sparse and precarious blood supply to the lower lumbar vertebrae, which is most commonly implicated in back pain [4-7,9,21,22]. In angiographic studies of cadaveric specimens, anastomotic networks that are thought to compensate for diminished flow between vertebrae were less prevalent with advancing age [9,21]. In addition, blood supply to the lumbar spine is further diminished in atherosclerotic disease [21]. Kauppila et al. showed that atherosclerosis of the abdominal aorta and smaller vessels such as the segmental arteries that supply the lumbar discs is associated with back pain [9,21]. Perhaps the most compelling study was the Framingham study, where individuals with aortic wall calcifications on X-ray were more likely to have evidence of disc degeneration and report back pain during a 25-year follow-up [5].

Hypercholesterolemia is a primary cardiovascular risk factor hypothesized to contribute to the development of lumbar disc degeneration [12]. The mechanism involves cholesterol contributing to atherosclerosis of the lumbar arteries, which supply the intervertebral discs [9,13,15,21-26]. According to the vascular hypothesis, the lumbar spine is thought to depend on blood supply, as other organs are, and therefore is susceptible to the deleterious consequences of arterial narrowing and occlusion [9,21]. Therefore, it is reasonable to infer that high serum cholesterol levels leading to atherosclerosis of the lumbar arteries may contribute significantly to degeneration.

Following the vascular theory, treatment for atherosclerosis might offer a new means for the management of degenerative conditions of the spine. Statins are amongst the most widely used medications in the treatment of hypercholesterolemia. Prior studies have shown the association between hypercholesterolemia and osteoarthritis [27]. However, the impact of statins in osteoarthritis remains unclear. Some studies have concluded that statin use has a positive impact on osteoarthritis progression [28-31], yet others have found no significant impact [32-36]. Studies examining the impact of statin use in degenerative conditions of the spine have primarily been in vivo and have shown that statins might slow the progression of degeneration and stimulate the repair of intervertebral discs [37-40]. A retrospective cohort study by Cheng et al. showed a significant reduction in spinal joint degeneration incidence in long-term high-dose statin users [41]. Despite the discourse, there is data to suggest hyperlipidemia impacts musculoskeletal degeneration. Management of hyperlipidemia should be investigated further to prevent degenerative changes of the spine.

Our findings are supportive of the vascular hypothesis as it relates to back pain. With the ubiquitous nature of back pain among Western populations, new strategies for managing this condition are warranted. This study suggests that early prevention or treatment of high serum cholesterol levels may potentially be useful in managing and preventing the development of back pain and lumbar disorders.

Limitations

While relevant variables were considered, there may be covariates that were not captured in the MEPS database and were therefore not assessed. The data in this survey is all self-reported. However, MEPS questionnaires are standardized and anonymous, which mitigates the risk for bias. Due to the nature of the survey, specific values for serum cholesterol, atherosclerotic genealogy, and physical activity, were not available, which limited our stratification of subjects. In addition, a more detailed examination of potential contributory confounders to the reporting of back pain would benefit any future study. An in-depth examination of the relationship between prior spine trauma and back pain could potentially influence findings. Moreover, another limitation factor comes from the fact that the survey respondents for back pain items were predominantly male, accounting for 91% of the subjects. Therefore, these data should be interpreted cautiously and future studies may be aimed at mitigating these limitations. 

## Conclusions

In this study, subjects with back pain were 34% more likely to have high serum cholesterol levels. The results of this study provide further evidence in support of the vascular theory for back pain. Hypercholesterolemia is a significant contributor to the development of cardiovascular disease. The specific link between hypercholesterolemia and back pain is an emerging area of interest. In order to better understand the relationship between hypercholesterolemia and back pain, more comprehensive and systematic studies are needed. Ultimately, further investigation could uncover new therapeutic approaches for managing back pain in high-cholesterol individuals and help to better understand its etiology. New strategies for the treatment of back pain may focus on targeting and preventing cardiovascular disease.
